# Association between the cumulative average triglyceride glucose-body mass index and cardiovascular disease incidence among the middle-aged and older population: a prospective nationwide cohort study in China

**DOI:** 10.1186/s12933-023-02114-w

**Published:** 2024-01-06

**Authors:** Fadong Li, Yue Wang, Boqun Shi, Shuaifeng Sun, Shen Wang, Shuo Pang, Xiaofan Wu

**Affiliations:** Department of Cardiology, Beijing Anzhen Hospital, Capital Medical University, Anzhen Road, Beijing, 100029 China

**Keywords:** Cardiovascular disease, Insulin resistance, TyG-BMI, CHARLS, Prospective cohort study

## Abstract

**Background:**

Findings from earlier research have established that insulin resistance (IR) is implicated in atherosclerosis progression, representing a noteworthy risk factor for cardiovascular disease (CVD). Recently, the triglyceride glucose-body mass index (TyG-BMI) has been introduced as a straightforward and robust alternative indicator for early detection of IR. Nevertheless, there is a scarcity of studies that have examined the capability of TyG-BMI for predicting incident CVD. Consequently, the core objective of this study was to determine whether the cumulative average TyG-BMI correlated with CVD incidence.

**Methods:**

All data was sourced from the China Health and Retirement Longitudinal Study (CHARLS). The exposure was the cumulative average TyG-BMI, determined by the average of TyG-BMI values for the baseline and follow-up investigations (Wave 1 in 2011, Wave 3 in 2015, respectively). The calculation of TyG-BMI involved a combination of triglyceride, fasting blood glucose, and body mass index. The primary outcome was incident CVD. Logistic regression analyses as well as restricted cubic spline (RCS) regression analyses were performed for examining the association between the cumulative average TyG-BMI and CVD incidence.

**Results:**

In all, 5,418 participants were enrolled in our analysis, with 2,904 (53.6%) being female, and a mean (standard deviation, SD) age of 59.6 (8.8) years. The mean (SD) cumulative average TyG-BMI among all participants was 204.9 (35.7). Totally, during a 4-year follow-up, 543 (10.0%) participants developed CVD. The fully adjusted logistic regression analysis revealed a significant association between the cumulative average TyG-BMI and incident CVD [odds ratio (OR), 95% confidence interval (CI): 1.168, 1.040–1.310, per 1 SD increase]. The RCS regression analysis displayed a positive, linear association of the cumulative average TyG-BMI with CVD incidence (P for overall = 0.038, P for nonlinear = 0.436).

**Conclusions:**

Our study revealed a noteworthy correlation between the cumulative average TyG-BMI and incident CVD among the middle-aged and older population. The cumulative average TyG-BMI emerges as a valuable tool that may enhance the primary prevention and treatment of CVD.

**Supplementary Information:**

The online version contains supplementary material available at 10.1186/s12933-023-02114-w.

## Introduction

Cardiovascular disease (CVD) continues to stand as a prominent contributor to morbidity and mortality on a global scale, placing a substantial burden on both healthcare systems and the well-being of individuals. Globally, the CVD burden has continued to rise over the past 30 years, with the prevalent cases of total CVD expanding by 92.3% (from 271 to 523 million, 1990–2019) and the number of deaths increasing by 53.7% (from 12.1 million to 18.6 million, 1990–2019) [1]. Notably, low-income and middle-income countries exhibited an even higher CVD incidence and related mortality when compared to high-income countries [2, 3]. Therefore, intensification of current strategies to identify high-risk individuals is needed to reduce CVD incidence and associated mortality rates further.

Insulin resistance (IR) refers to a pathophysiological disorder distinguished by defective insulin regulation of glucose metabolism in tissue cells, and it is considered a crucial element contributing to the development of type 2 diabetes and CVD [4–6]. To date, the hyperinsulinemic- euglycemic clamp (HEC) remains the gold standard for assessing IR, but this approach entails extensive labor, high costs, and hence unfeasible for broad clinical applications [7]. Recently, the triglyceride glucose-body mass index (TyG-BMI) has been introduced as a straightforward and robust alternative indicator for early detection of IR, revealing its potential utility in clinical settings [8]. A prior study based on a nationwide survey demonstrated that, when compared to the triglyceride-glucose index (TyG) as well as its correlated indices, the TyG-BMI exhibited the highest predictive capability for IR [9]. The TyG-BMI has been well studied in associations with diverse diseases, encompassing diabetes, non-alcoholic fatty liver disease (NAFLD), and hypertension [10–12].

However, there were limited studies shedding light on the relationship between the TyG-BMI and CVD incidence, which requires further exploration and validation. With regards to the above, we utilized data from the China Health and Retirement Longitudinal Study (CHARLS), aiming to investigate the association between the cumulative average TyG-BMI and CVD incidence among the population aged 45 years or older.

## Methods

### Data source and study population

**Fig. 1:**
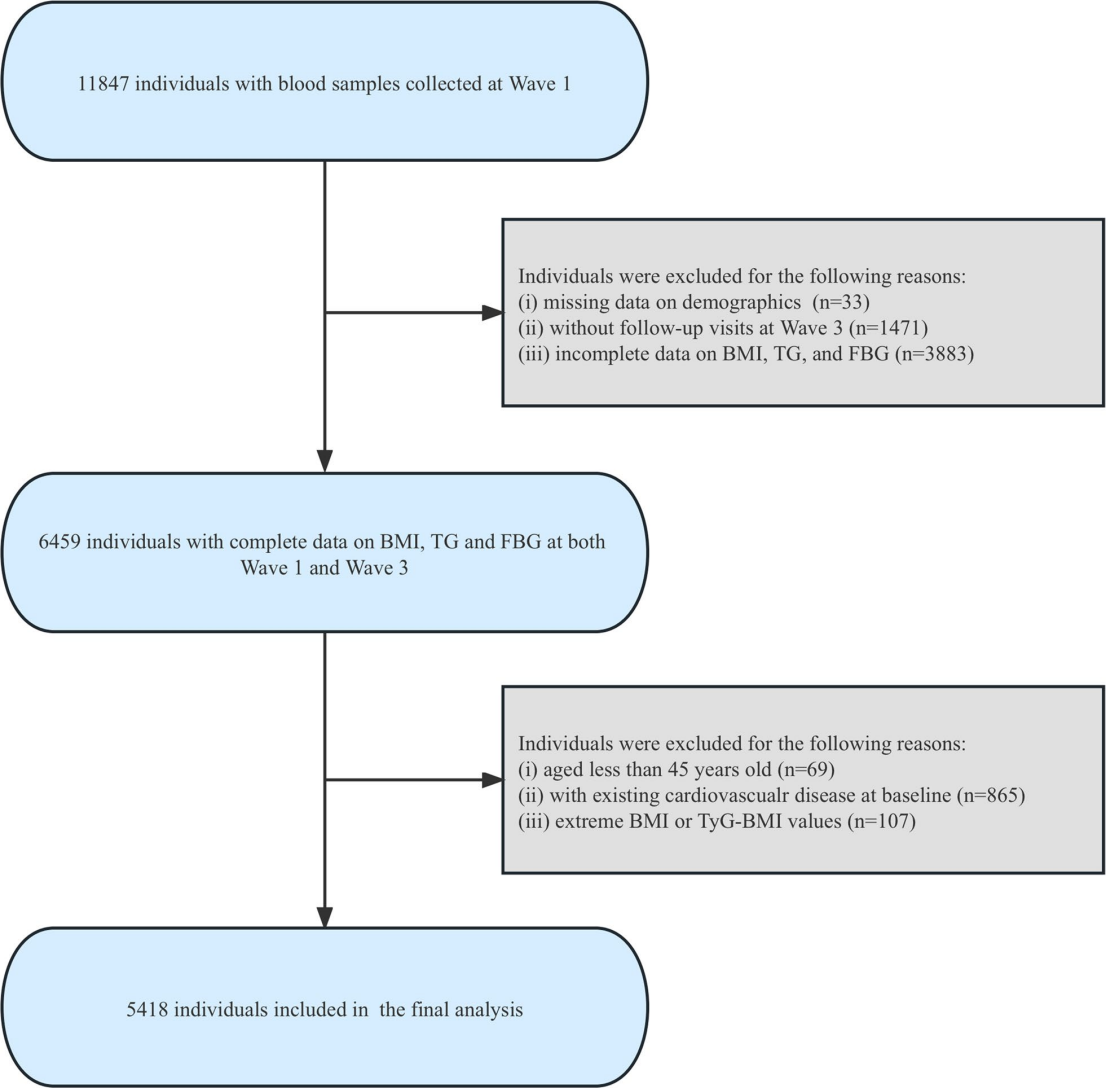
Flowchart of the study population. BMI, body mass index; TG, triglyceride; FBG, fasting blood glucose; TyG-BMI, triglyceride glucose-body mass index

All data was sourced from the CHARLS cohort, a widely recognized longitudinal study that focuses on individuals middle-aged and older (≥ 45 years), and which is representative of the national population. The initial nationwide survey of CHARLS was conducted in 2011 (Wave 1), successfully interviewing 17,708 individuals in 10,257 households across 150 counties/districts and 450 villages/resident committees in China. Following the baseline wave, subsequent follow-up surveys were scheduled at biennial intervals, including 2013, 2015, 2018 for Wave 2, Wave 3, Wave 4, respectively. Blood samples of participants were obtained at Wave 1 and Wave 3 as well (11,847 and 13,420 participants respectively). Detailed information on sampling method, anthropometric measures, and blood biomarker information of CHARLS has been documented in other publications before [13, 14]. Considering available blood examination data, the datasets for Wave 1 (baseline, 2011) and Wave 3 (follow-up, 2015) were extracted. We first included 11,847 participants who had blood sample collected at Wave 1, and we excluded those without demographics data (n = 33), without follow-up visits at Wave 3 (n = 1,471), or without complete data on body mass index (BMI), triglyceride (TG) or fasting blood glucose (FBG) at Wave 1 or Wave 3 (n = 3883). Participants younger than 45 years old (n = 69), and participants with extreme values of BMI (< 15 or > 55 kg/m^2^) or TyG-BMI (less or more than 3 standard deviations from the mean) (n = 107) were also excluded in our analysis. Furthermore, due to the design of the current study, participants with established diagnosis of CVD at baseline survey (Wave 1) were excluded (n = 865). Ultimately, there were a total of 5418 eligible participants enrolled in our analysis (Fig. 1).

The ethical review in the approval process of CHARLS was conducted by the Peking University institutional review board. All study participants granted formal written consent for their involvement. The principles outlined in the Strengthening the Reporting of Observational Studies in Epidemiology (STROBE) were followed in the current study [15].

### Data assessment and definitions

#### Assessment of exposure

The exposure was the cumulative average TyG-BMI, employed to represent the longstanding status of IR. The calculation of TyG-BMI involved the formula as follows: ln (TG (mg/dL) × FBG (mg/dL)/2) × BMI [8]. The cumulative average TyG-BMI was calculated using the following formula: (TyG-BMI_2011_ + TyG-BMI_2015_)/2.

#### Assessment of outcomes

Incident CVD was the primary outcome of this study. At each survey of CHARLS, participants were inquired “Have you been diagnosed with a stroke/heart condition (including heart attack, coronary heart disease, angina, congestive heart failure or other heart problems) by a doctor?” and “Are you now undergoing any of the following treatments (Taking Chinese Traditional Medicine/Taking Western Modern Medicine/Other treatments/None of the Above) to treat stroke/heart condition or its complications?” during questionnaire surveys. Similar to previous studies [16–18], participants who self-reported “yes” for receiving a diagnosis of a heart condition or stroke from a doctor or those who indicated specific treatment for a heart condition or stroke, were defined as individuals with CVD.

### Data collection

The following data was collected in this study: (i) demographics: gender, age, education level, residence (hukou), and marital status; (ii) body measurements: systolic blood pressure (SBP), diastolic blood pressure (DBP), and BMI; (iii) lifestyle information: smoking status and alcohol consumption status; (iv) medical history: dyslipidemia, hypertension, diabetes, kidney disease, liver disease, lipid-lowering treatment, antihypertensive treatment, and hypoglycaemic treatment; (v) laboratory examinations: glycosylated hemoglobin (HbA1c), FBG, TG, total cholesterol (TC), high-density lipoprotein cholesterol (HDL-c), low-density lipoprotein cholesterol (LDL-c). Participants who self-reported a history of hypertension or received any specific treatment for hypertension, and those who had a mean SBP ≥ 140 mmHg or a mean DBP ≥ 90 mmHg at baseline, were defined as individuals with hypertension [19]. Participants that self-reported a history of diabetes, underwent any hypoglycaemic treatment or had a FBG ≥ 7.0 mmol/L (126 mg/dL) at baseline, were regarded as having diabetes [20]. Other medical conditions were determined by a self-reported history or undergoing any specific treatment.

### Handling of missing variables

Additional file 1: Figure S1 illustrated the distribution of variables with missing data in our study. Aiming to maintain the largest possible sample size, we employed the multiple imputation method for addressing missing variables despite of the small proportion of missing data.

### Statistical analysis

The study population (n = 5418) was classified in four groups based on quartiles of the cumulative average TyG-BMI [from Quartile 1 (Q1) to Q4]. To present participants’ baseline characteristics, means ± standard deviations (SD) were provided for continuous variables, and one-way-ANOVA test was utilized for comparing differences among groups. While for categorical variables, numbers (percentages) were presented, and Pearson chi-squared test was utilized to analyze inter-group differences.

To evaluate the relationship between the cumulative average TyG-BMI and CVD incidence, the univariable and multivariable logistic regression models were established, and odds ratios (OR) with their 95% confidence intervals (95% CI) were presented. In Model 1, adjustments for age and gender were included. In Model 2, adjustments for age, gender, smoking status, alcohol consumption status, SBP, DBP, HbA1c, TC, HDL-c, LDL-c were included. In Model 3, adjustments for all covariates comprised in Model 2, in addition to residence, education level, marital status, hypertension, dyslipidemia, diabetes, liver disease, kidney disease, antihypertensive treatment, lipid-lowering treatment and hypoglycaemic treatment were included. In the multicollinearity test, the variance inflation factor (VIF) [21] for each variable included in our analysis was determined, which was all below 5 (Additional file 3: Table S1), suggesting no evidence of significant multicollinearity. Additionally, a multivariable adjusted (fully adjusted) restricted cubic spline (RCS) logistic regression analysis (choosing 4 knots, 5th, 35th, 65th and 95th percentiles, respectively) was conducted, for examining the linearity and the dose–response relationship between the cumulative average TyG-BMI and CVD incidence. Furthermore, in order to detect the potential modifications, a variety of subgroup analyses and interaction analyses were performed. Participants were stratified into diverse subgroups, including age (< 60 vs. ≥ 60 years), gender, smoking status, drinking status, as well as presence of hypertension, dyslipidemia, and diabetes. All statistical analyses were performed with R software version 4.3.1 (http://www.R-project.org/). A two-tailed P value < 0.05 was considered statistically significant.

### Sensitivity analysis

The study results were further validated through conducting a set of sensitivity analyses. Firstly, we fitted a logistic regression model excluding participants with any missing value of variables to eliminate the possible impact of missing values to the primary outcome. Secondly, we reanalyzed the data after excluding participants that already had CVD at Wave 2 (2013) to test whether the relatively short-term onset of CVD had an effect on the primary outcome. Thirdly, we established a well-matched cohort at baseline utilizing the 1:1 propensity score matching, with nearest-neighbor matching, no replacement and a caliper width of 0.01. An acceptable balance between groups (Q1-Q2 vs. Q3-Q4) was determined by a standardized mean difference (SMD) below 0.10.

## Results

### Baseline characteristics of participants

The flowchart that illustrated the study population screening was presented in Fig. 1. There were 5418 participants enrolled in our study. Among the 5418 participants enrolled in our final analysis, 2904 were female (53.6%), while the mean (SD) age was 59.6 (8.8) years.

The baseline characteristics of enrolled participants, categorized by quartiles of the cumulative average TyG-BMI, were outlined in Table 1. Among all included participants, the mean (SD) cumulative average TyG-BMI was 204.9 (35.7). Contrasted with those in the lowest quartile group (Q1), participants in groups with higher levels of the cumulative average TyG-BMI (Q2-Q4) were younger, exhibited a greater proportion of females and urban residents, featured greater values of SBP, DBP, TC, LDL-c, HbA1c and lower values of HDL-c, and showed a greater frequency of hypertension, dyslipidemia and diabetes (all P-value < 0.05). Moreover, no statistically significant differences in the prevalence of liver or kidney disease were observed across all groups.

**Table 1:** Baseline characteristics of individuals classified by quartiles of the cumulative average TyG-BMI

Characteristics	Overall	Quartiles of the cumulative average TyG-BMI				
		Quartile 1	Quartile 2	Quartile 3	Quartile 4	P-value
n	5418	1355	1354	1354	1355	
Gender (Female)	2,904 (53.6%)	556 (41.0%)	693 (51.2%)	777 (57.4%)	878 (64.8%)	< 0.001
Age, years	59.6 ± 8.8	62.2 ± 9.5	59.9 ± 8.6	58.8 ± 8.4	57.7 ± 8.0	< 0.001
Residence (Urban)	747 (13.8%)	120 (8.9%)	158 (11.7%)	230 (17.0%)	239 (17.6%)	< 0.001
Education level						< 0.001
Elementary school or below	3,833 (70.7%)	1,026 (75.7%)	978 (72.2%)	918 (67.8%)	911 (67.2%)	
Middle school	1,454 (26.8%)	307 (22.7%)	346 (25.6%)	393 (29.0%)	408 (30.1%)	
College or above	131 (2.4%)	22 (1.6%)	30 (2.2%)	43 (3.2%)	36 (2.7%)	
Current married	4,838 (89.3%)	1,165 (86.0%)	1,194 (88.2%)	1,237 (91.4%)	1,242 (91.7%)	< 0.001
SBP, mmHg	129.9 ± 25.0	124.9 ± 20.7	127.5 ± 24.6	130.4 ± 22.2	136.9 ± 30.0	< 0.001
DBP, mmHg	75.5 ± 12.1	71.9 ± 11.4	73.8 ± 11.8	76.4 ± 11.6	79.8 ± 11.9	< 0.001
BMI, kg/m^2^	23.4 ± 3.4	19.6 ± 1.6	22.1 ± 1.4	24.2 ± 1.6	27.5 ± 2.4	< 0.001
Smoking status						< 0.001
Never	3,314 (61.2%)	656 (48.4%)	804 (59.4%)	887 (65.5%)	967 (71.4%)	
Former	449 (8.3%)	110 (8.1%)	103 (7.6%)	121 (8.9%)	115 (8.5%)	
Current	1,655 (30.5%)	589 (43.5%)	447 (33.0%)	346 (25.6%)	273 (20.1%)	
Drinking status						< 0.001
Never	3,174 (58.6%)	702 (51.8%)	778 (57.5%)	798 (58.9%)	896 (66.1%)	
Former	310 (5.7%)	87 (6.4%)	68 (5.0%)	83 (6.1%)	72 (5.3%)	
Current	1,934 (35.7%)	566 (41.8%)	508 (37.5%)	473 (34.9%)	387 (28.6%)	
Dyslipidemia	414 (7.6%)	34 (2.5%)	60 (4.4%)	118 (8.7%)	202 (14.9%)	< 0.001
Hypertension	2,034 (37.5%)	336 (24.8%)	420 (31.0%)	548 (40.5%)	730 (53.9%)	< 0.001
Diabetes	819 (15.1%)	108 (8.0%)	152 (11.2%)	200 (14.8%)	359 (26.5%)	< 0.001
Liver disease	189 (3.5%)	54 (4.0%)	40 (3.0%)	54 (4.0%)	41 (3.0%)	0.261
Kidney disease	303 (5.6%)	82 (6.1%)	82 (6.1%)	65 (4.8%)	74 (5.5%)	0.433
Lipid-lowering treatment	220 (4.1%)	14 (1.0%)	29 (2.1%)	61 (4.5%)	116 (8.6%)	< 0.001
Antihypertensive treatment	855 (15.8%)	86 (6.3%)	139 (10.3%)	231 (17.1%)	399 (29.4%)	< 0.001
Hypoglycemic treatment	255 (4.7%)	31 (2.3%)	40 (3.0%)	61 (4.5%)	123 (9.1%)	< 0.001
FBG, mg/dL	109.4 ± 34.4	101.6 ± 21.8	105.5 ± 27.0	109.1 ± 34.6	121.3 ± 46.1	< 0.001
HbA1c, %	5.3 ± 0.8	5.1 ± 0.6	5.2 ± 0.7	5.2 ± 0.7	5.5 ± 1.0	< 0.001
TG, mg/dL	131.4 ± 102.6	84.4 ± 41.3	108.5 ± 75.2	134.3 ± 83.4	198.5 ± 143.4	< 0.001
TC, mg/dL	193.4 ± 37.7	184.7 ± 35.6	191.1 ± 36.4	195.0 ± 36.7	202.8 ± 39.8	< 0.001
HDL-c, mg/dL	51.5 ± 15.3	59.8 ± 16.4	54.0 ± 14.4	49.2 ± 12.8	42.8 ± 12.1	< 0.001
LDL-c, mg/dL	116.2 ± 34.6	109.7 ± 30.6	116.9 ± 32.7	119.2 ± 34.1	118.9 ± 39.5	< 0.001
TyG	8.7 ± 0.7	8.3 ± 0.5	8.5 ± 0.5	8.7 ± 0.6	9.2 ± 0.7	< 0.001
Cumulative average TyG-BMI	204.9 ± 35.7	162.2 ± 11.6	190.0 ± 6.8	214.0 ± 7.7	253.5 ± 19.1	< 0.001

SBP, systolic blood pressure; DBP, diastolic blood pressure; BMI, body mass index; FBG, fasting blood glucose; HbA1c, glycosylated hemoglobin A1c; TG, triglyceride; TC, total cholesterol; HDL‐c, high‐density lipoprotein cholesterol; LDL-c, low-density lipoprotein cholesterol; TyG, triglyceride glucose index; TyG-BMI, triglyceride glucose-body mass index

In addition, the distribution of the cumulative average TyG-BMI in this study was visualized in Additional file 2: Figure S2. Additional file 3: Table S2 provided a summary of participants’ baseline characteristics, which were categorized according to the presence or absence of incident CVD.

### Association between the cumulative average TyG-BMI and cardiovascular disease incidence

**Fig. 2:**
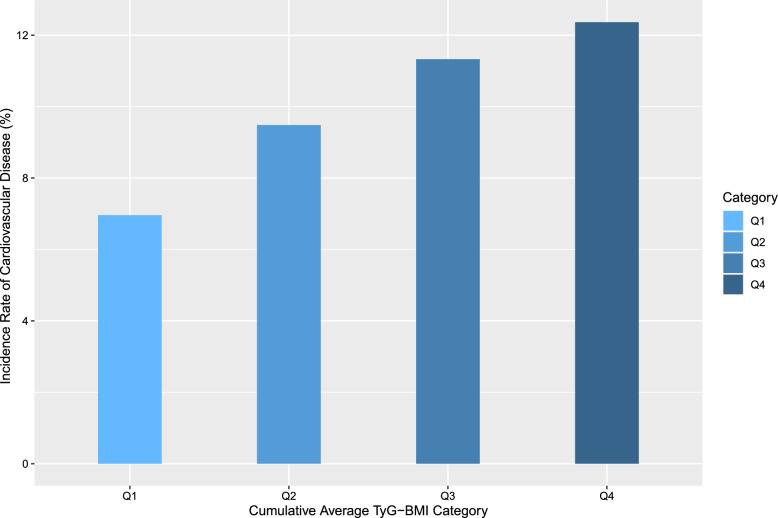
Incidence rates of CVD categorized by quartiles of the cumulative average TyG-BMI. Q1, Quartile 1; Q2, Quartile 2; Q3, Quartile 3; Q4, Quartile 4; TyG-BMI, triglyceride glucose-body mass index

In total, 543 (10.0%) participants developed CVD during a 4-year follow-up (Wave 1 in 2011 to Wave 3 in 2015). As depicted in Fig. 2, the incidence rates of CVD increased progressively across quartiles of the cumulative average TyG-BMI (from Q1 to Q4), with 94 (6.9%), 128 (9.5%), 154 (11.4%), and 167 (12.3%) cases observed in four groups of participants, respectively (Table 2).

**Table 2:** Association between the cumulative average TyG-BMI and CVD incidence

Cumulative Average TyG-BMI	Quartiles					Continuous
	Quartile 1	Quartile 2	Quartile 3	Quartile 4	P for trend	Per 1 SD increase
Median	164.6	190.3	213.5	249.1	–	–
Cases, n (%)	94 (6.9%)	128 (9.5%)	154 (11.4%)	167 (12.3%)	–	–
Crude, OR (95% CI)	Reference	1.401 (1.062–1.852)	1.722 (1.319–2.258)	1.886 (1.450–2.465)	< 0.001	1.255 (1.151–1.368)
Model 1, OR (95% CI)	Reference	1.504 (1.137–1.995)	1.900 (1.448–2.506)	2.133 (1.625–2.816)	< 0.001	1.310 (1.197–1.432)
Model 2, OR (95% CI)	Reference	1.505 (1.131–2.011)	1.880 (1.406–2.524)	2.066 (1.501–2.856)	< 0.001	1.309 (1.173–1.460)
Model 3, OR (95% CI)	Reference	1.428 (1.070–1.913)	1.635 (1.215–2.208)	1.560 (1.118–2.185)	0.018	1.168 (1.040–1.310)

Model 1, adjusted for age and gender; Model 2, adjusted for age, gender, smoking status, drinking status, SBP, DBP, HbA1c, TC, HDL-c, LDL-c; Model 3, adjusted for variables included in Model 2 and residence (hukou), education level, marital status, hypertension, dyslipidemia, diabetes, liver disease, kidney disease, antihypertensive treatment, lipid-lowering treatment and hypoglycaemic treatment

TyG-BMI, triglyceride glucose-body mass index; OR, odds ratio; CI, confidence interval; SD, standard deviation

After adjustments for multiple covariates, the fully adjusted logistic regression model indicated that higher levels of the cumulative average TyG-BMI (Q2-Q4) increased the odds ratios for incident CVD in contrast to Q1 (OR, 95% CI 1.428, 1.070–1.913, Q2; OR, 95% CI 1.635,1.215–2.208, Q3; OR, 95% CI 1.560,1.118–2.185, Q4) (Table 2). Consistently, when considered as a continuous variable, per 1 SD rise in the cumulative average TyG-BMI was significantly associated with incident CVD (OR, 95% CI 1.168, 1.040–1.310). Moreover, the logistic regression analyses, examining the correlation between the cumulative average TyG-BMI and the components of CVD (heart condition or stroke), revealed that the cumulative average TyG-BMI was significantly related to incident heart condition (OR, 95% CI 1.519, 1.111–2.085, Q2; OR, 95% CI 1.791, 1.301–2.479, Q3; OR, 95% CI 1.790, 1.252–2.571, Q4; OR, 95% CI 1.225,1.084–1.384, per 1 SD increase), but not related to incident stroke, which was shown in Additional file 3: Table S3.

**Fig. 3:**
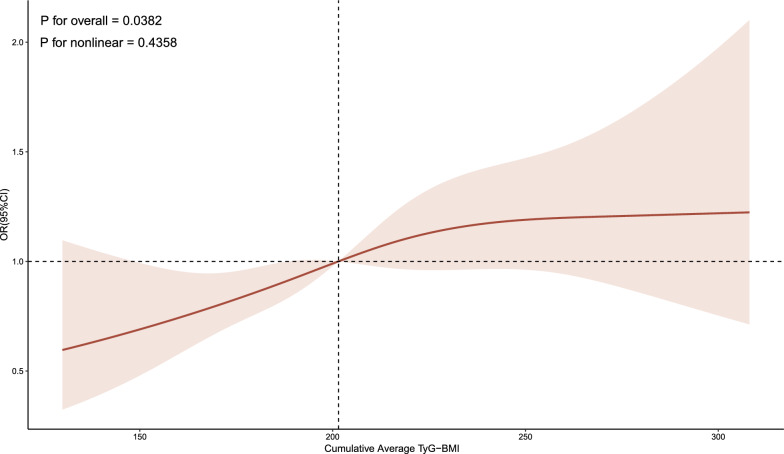
Association between the cumulative average TyG-BMI and incident CVD. The model was adjusted for age, gender, smoking status, drinking status, SBP, DBP, HbA1c, TC, HDL-c, LDL-c, residence (hukou), education level, marital status, hypertension, dyslipidemia, diabetes, liver disease, kidney disease, antihypertensive treatment, lipid-lowering treatment and hypoglycaemic treatment. SBP, systolic blood pressure; DBP, diastolic blood pressure; HbA1c, glycosylated hemoglobin A1c; TC, total cholesterol; HDL‐c, high‐density lipoprotein cholesterol; LDL-c, low-density lipoprotein cholesterol; TyG-BMI, triglyceride glucose-body mass index; OR, odds ratio; CI, confidence interval

**Fig. 4:**
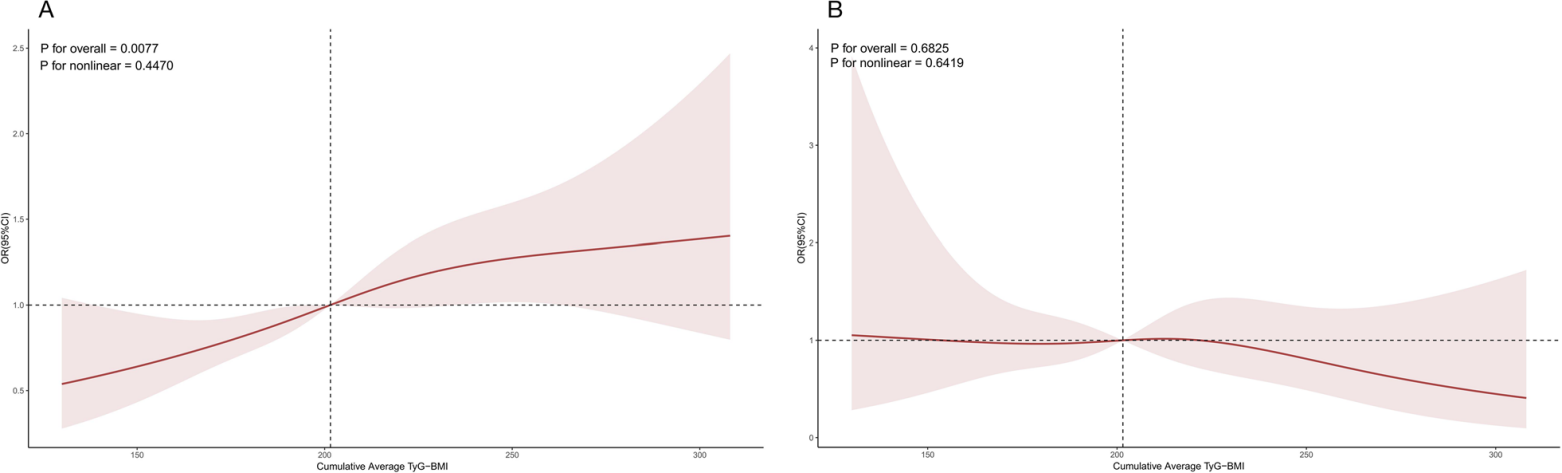
Association between the cumulative average TyG-BMI and incident heart condition (A) and incident stroke (B). The model was adjusted for age, gender, smoking status, drinking status, SBP, DBP, HbA1c, TC, HDL-c, LDL-c, residence (hukou), education level, marital status, hypertension, dyslipidemia, diabetes, liver disease, kidney disease, antihypertensive treatment, lipid-lowering treatment and hypoglycaemic treatment. SBP, systolic blood pressure; DBP, diastolic blood pressure; HbA1c, glycosylated hemoglobin A1c; TC, total cholesterol; HDL‐c, high‐density lipoprotein cholesterol; LDL-c, low-density lipoprotein cholesterol; TyG-BMI, triglyceride glucose-body mass index; OR, odds ratio; CI, confidence interval

The fully adjusted RCS regression model indicated a positive, linear correlation between the cumulative average TyG-BMI and incident CVD (P for overall = 0.038, P for nonlinear = 0.436) (Fig. 3). Furthermore, the RCS regression model indicated that a linear relationship between the cumulative average TyG-BMI and incident heart condition (P for overall = 0.008, P for nonlinear = 0.447) (Fig. 4). Nonetheless, the cumulative average TyG-BMI did not reveal a notable association with incident stroke in our analysis (P for overall = 0.683) (Fig. 4).

### Subgroup analysis

**Fig. 5:**
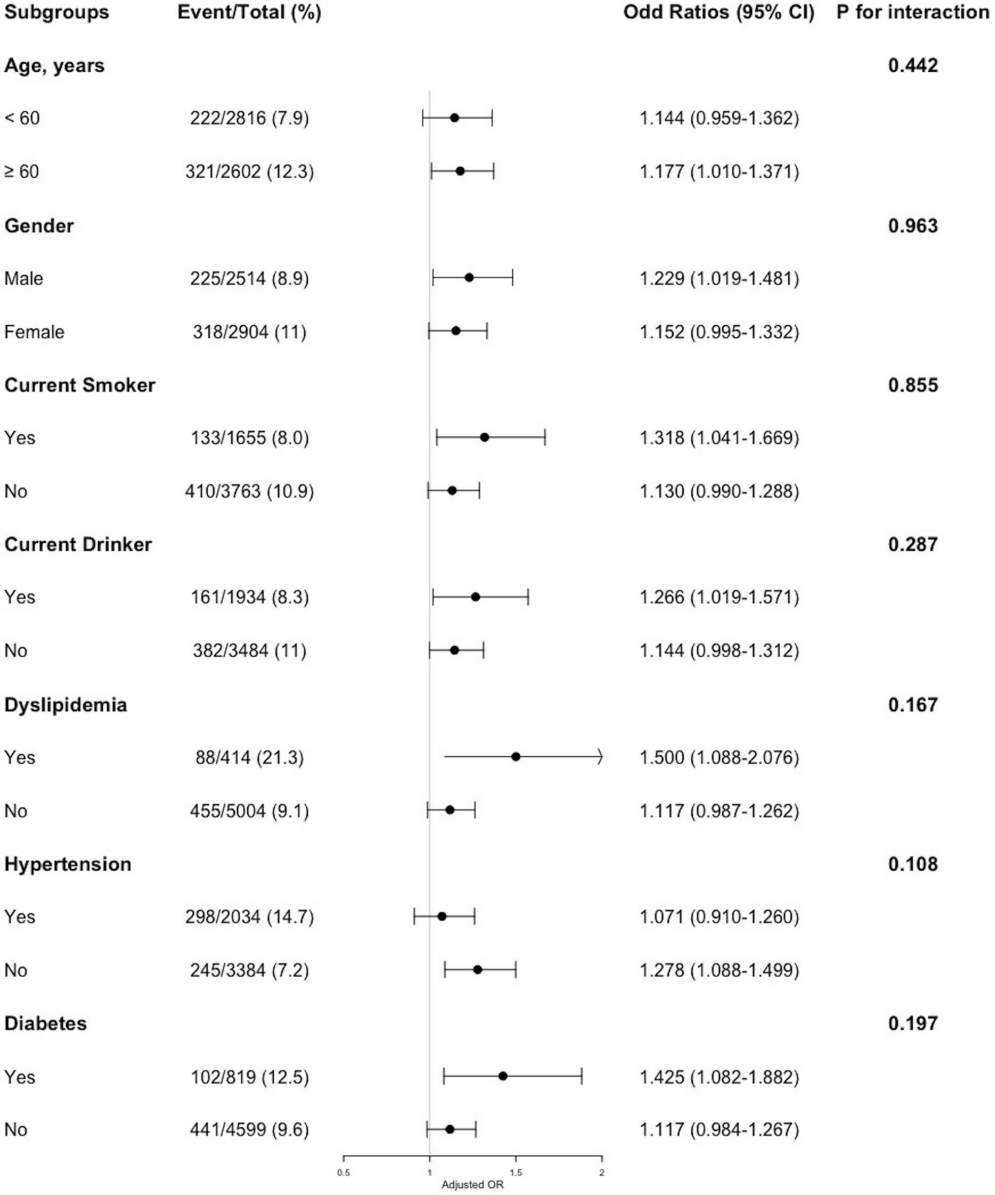
Subgroup analyses of the association between the cumulative average TyG-BMI and CVD incidence. The model was adjusted for age, gender, smoking status, drinking status, SBP, DBP, HbA1c, TC, HDL-c, LDL-c, residence (hukou), education level, marital status, hypertension, dyslipidemia, diabetes, liver disease, kidney disease, antihypertensive treatment, lipid-lowering treatment and hypoglycaemic treatment (excluding the variable for subgroup stratification). SBP, systolic blood pressure; DBP, diastolic blood pressure; HbA1c, glycosylated hemoglobin A1c; TC, total cholesterol; HDL‐c, high‐density lipoprotein cholesterol; LDL-c, low-density lipoprotein cholesterol; OR, odds ratio; CI, confidence interval

For the purpose of further investigating the relationship between the cumulative average TyG-BMI and CVD incidence, a series of subgroups analyses were conducted. As shown in Fig. 5, none of the subgroups including age, gender, current smoker, current drinker, and prevalence of dyslipidemia, hypertension or diabetes, profoundly changed the relationship between the cumulative average TyG-BMI and CVD incidence (all P for interaction > 0.05).

### Sensitivity analysis

Consistent outcomes were observed when various methods were employed to validate the robustness of the primary results in our study.

To begin with, the positive correlation between the cumulative average TyG-BMI and the incidence of CVD was consistent after removing participants with any missing value of covariates in the fully adjusted model (OR, 95% CI 1.489, 1.111–2.003, Q2; OR, 95% CI 1.644, 1.215–2.233, Q3; OR, 95% CI 1.582,1.127–2.228, Q4; OR, 95% CI 1.169, 1.040–1.314, per 1 SD increase) (Additional file 3: Table S4). Next, after excluding individuals who had already developed CVD at Wave 2 (2013), the result aligned with the primary analysis (OR, 95% CI 1.429, 1.019–2.017, Q2; OR, 95% CI 1.630, 1.153–2.319, Q3; OR, 95% CI 1.547,1.051–2.290, Q4; OR, 95% CI 1.191, 1.043–1.360, per 1 SD increase) (Additional file 3: Table S5). At last, after performing 1:1 propensity score matching, 1,355 pairs of baseline-matched individuals in Q1-Q2 and Q3-Q4 groups were confirmed (Additional file 3: Table S6). Consistently, there exhibited a strong correlation between the cumulative average TyG-BMI and the incidence of CVD (OR, 95% CI 1.337, 1.042–1.719, Q3-Q4 vs. Q1-Q2; OR, 95% CI 1.190, 1.054–1.342, per 1 SD increase) (Additional file 3: Table S7).

## Discussion

In the current prospective, nationwide, longitudinal cohort study involving the population aged 45 years and above in China, encompassing 5,418 participants with an extended 4-year follow-up duration, a notable relationship between the cumulative average TyG-BMI and incident CVD was observed after adjusting for confounders. Additionally, the fully adjusted RCS regression analysis displayed a positive, linear correlation between the cumulative average TyG-BMI and the incidence of CVD. As far as we can ascertain, the current study is the primary nationwide prospective cohort study targeting the population aged 45 years and above, with the aim of examining the correlation between the cumulative average TyG-BMI and incident CVD. Our findings may offer novel insights that could contribute to advancing strategies for the prevention of CVD.

CVD continues to stand as a pervasive global health concern, contributing significantly to the burden of healthcare systems worldwide. Additionally, it is imperative to note that low-income and middle-income countries exhibited more substantial CVD burden in comparison to high-income countries. IR is a pathophysiological condition that is marked by impaired insulin regulation of glucose metabolism in peripheral cells (prominently in skeletal muscle tissue, adipose tissue, and hepatic tissue) [6]. Evidence from previous studies proved that IR contributed to the pathogenesis of atherosclerosis and was recognized as a notable risk factor for CVD [22–25]. The HEC, which is the gold standard to detect IR, requires sophisticated and costly technique, making it impractical in clinical settings [7]. Therefore, there is an urgent need to identify a reliable alternative indicator for a more widespread assessment of IR.

Recently, the TyG-BMI, a novel index which involves TG, FBG, and BMI, was suggested as a simple yet powerful surrogate indicator for IR in clinical settings [8, 9, 26], and was proven to be associated with diverse medical conditions. Previous studies indicated a robust correlation between the TyG-BMI and NAFLD [11, 27, 28]. And it was revealed that higher TyG-BMI was linked with elevated risks of pre-diabetes, diabetes, and progression from pre-diabetes to diabetes [10, 29–31]. The TyG-BMI was also considered as a significant predictive factor in the development of hypertension [12, 32, 33]. A prior investigation observed that the TyG-BMI significantly correlated with ischemic stroke, suggesting its potential in enhancing the stratification of ischemic stroke risk [34]. Moreover, recent research has revealed a notable association between the TyG-BMI and the severity of coronary artery lesion [35], as well as its valuable predictive ability for major adverse cardiovascular events in patients receiving percutaneous coronary intervention [36, 37].

To date, restricted investigations have evaluated the relationship between the TyG-BMI and CVD risk. A prior observational study indicated a notable relationship between increased TyG-BMI and a heightened 10-year atherosclerotic cardiovascular disease (ASCVD) risk [38], yet it had certain limitations, including its cross-sectional design and the use of Pooled Cohort Equations to estimate ASCVD risk. Utilizing data from a large national longitudinal survey (CHARLS) with a 4-year follow-up, our study revealed a noteworthy correlation between the cumulative average TyG-BMI and the incidence of CVD. In addition, in the further analysis for exploring the association between the cumulative average TyG-BMI and the components of CVD, a notable correlation between the cumulative average TyG-BMI and heart condition was identified. However, no significant correlation was showed between the cumulative average TyG-BMI and stroke in our analysis, which was inconsistent with the prior study [34]. It cannot be dismissed that the limited number of incident stroke cases may have hindered our capacity to observe such an association. Furthermore, we observed that none of the subgroups (including age, gender, current smoker, current drinker, and prevalence of dyslipidemia, hypertension or diabetes) exhibited a significant modification to the relationship between the cumulative average TyG-BMI and CVD incidence, which manifests the applicability of our findings to the majority of individuals. Our findings significantly contributed to illuminating the relationship between the cumulative average TyG-BMI and incident CVD, highlighting its value as an economical and valuable early indicator for recognizing individuals prone to developing CVD.

The strengths of the current study comprised the abundant, credible medical data of CHARLS, the prospective nationwide cohort with extended follow-up, and thorough control for potential cardiovascular risk factors. More importantly, instead of baseline TyG-BMI, the cumulative average TyG-BMI was utilized in our study, which could represent the longstanding status of IR. Also, comprehensive subgroup analyses and sensitivity analyses in the current study bolstered the credibility of our assessment regarding the correlation between the cumulative average TyG-BMI and incident CVD.

Nonetheless, we have acknowledged some limitations inherent to the current study. First of all, due to the fact that blood samples were only obtained at Wave 1 (2011) and Wave 3 (2015), the cumulative average TyG-BMI was determined by the mean value of the TyG-BMI from these two waves. Consequently, we could not detect more specific changes of the TyG-BMI over time. Secondly, since CHARLS does not provide information on more accurate methods for diagnosing CVD, such as angiography, we defined the outcome of CVD as being diagnosed by a doctor or receiving treatment, which may result in a potential slight discrepancy with the actual incidence of CVD cases. However, the method employed in our study still ensures reasonably accurate identification of CVD cases, aligning with the approach used in many previous studies. Thirdly, taking into account the fact that this is an observational study, complete exclusion of residual confounding effects was not feasible, despite our efforts to control for potential confounders to the fullest extent possible. Finally, it's worth noting that the current study focused exclusively on the Chinese population aged 45 years and above. Further exploration is essential to verify the generalizability of our main findings to populations of diverse nationalities and a wider age range.

## Conclusions

The current study based on CHARLS indicated a significant association of the cumulative average TyG-BMI with incident CVD at a 4-year follow up. It should be noted that maintaining TyG-BMI at a relatively low level may enhance the primary prevention of CVD. Due to the easy availability, the cumulative average TyG-BMI is capable of serving as a valuable indicator to risk-stratify the middle-aged and older population for providing more personalized prevention or treatment of CVD.

### Supplementary Information

**Additional file 1: Figure S1.** Distribution of variables with missing data.

**Additional file 2:  Figure S2.** Distribution of the cumulative average TyG-BMI.

**Additional file 3: Table S1.** Collinearity Statistics. **Table S2.** Baseline characteristics of individuals classified by outcome. **Table S3.** Association between the cumulative average TyG-BMI and the components of CVD. **Table S4.** Association between the cumulative average TyG-BMI and CVD incidence after excluding individuals with any missing value. **Table S5.** Association between the cumulative average TyG-BMI and CVD incidence after excluding individuals with CVD at Wave 2. **Table S6.** Baseline characteristics before and after 1:1 propensity score matching. **Table S7.** Association between the cumulative average TyG-BMI and CVD incidence after 1:1 propensity score matching.

## Data Availability

The datasets generated and/or analysed during the current study are available in the China Health and Retirement Longitudinal Study repository [http://charls.pku.edu.cn].

## Abbreviations

IR: Insulin resistance
CVD: Cardiovascular disease
TyG-BMI: Triglyceride glucose-body mass index
CHARLS: China health and retirement longitudinal study
TG: Triglyceride
FBG: Fasting blood glucose
BMI: Body mass index
RCS: Restricted cubic spline
SD: Standard deviation
OR: Odds ratio
CI: Confidence interval
HEC: Hyperinsulinemic-euglycemic clamp
NAFLD: Non-alcoholic fatty liver disease
STROBE: Strengthening the reporting of observational studies in epidemiology
SBP: Systolic blood pressure
DBP: Diastolic blood pressure
HbA1c: Glycosylated hemoglobin
TC: Total cholesterol
HDL-c: High-density lipoprotein cholesterol
LDL-c: Low-density lipoprotein cholesterol
Q1: Quartile 1
Q2: Quartile 2
Q3: Quartile 3
Q4: Quartile 4
VIF: Variance inflation factor
SMD: Standardized mean difference
ASCVD: Atherosclerotic cardiovascular disease
