# Supplementary figures and images for: Association between the cumulative average triglyceride glucose-body mass index and cardiovascular disease incidence among the middle-aged and older population: a prospective nationwide cohort study in China
Source: Cardiovasc Diabetol. 2024 Jan 6;23:16. doi: 10.1186/s12933-023-02114-w (PMC10771655; doi:10.1186/s12933-023-02114-w)

| Age Group | Proportion (Yes) |
|-----------|------------------|
| 18-24     | 0.0005           |
| 25-34     | 0.0015           |
| 35-44     | 0.0025           |
| 45-54     | 0.0035           |
| 55-64     | 0.0045           |
| 65-74     | 0.0055           |
| 75-84     | 0.0065           |
| 85+       | 0.0075           |

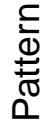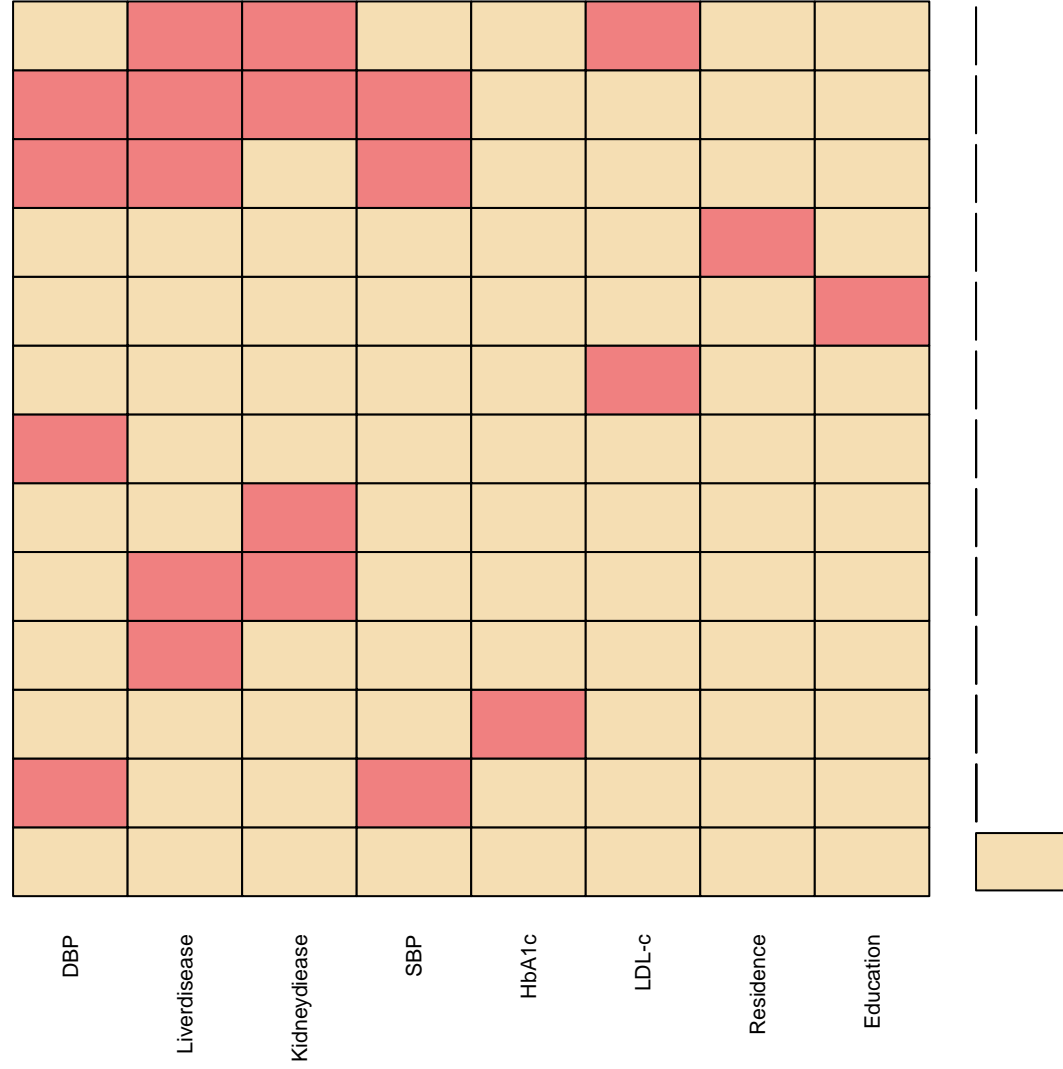

Supplement: Supplementary file 1 — Additional file 1: Figure S1. Distribution of variables with missing data. [file 12933_2023_2114_MOESM1_ESM.pdf]

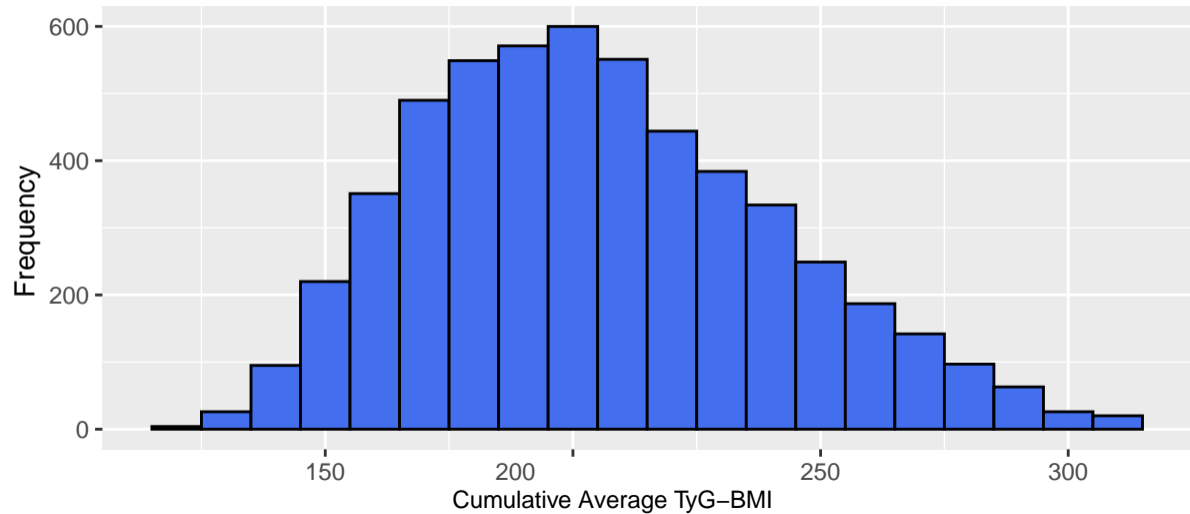

Supplement: Supplementary file 2 — Additional file 2: Figure S2. Distribution of the cumulative average TyG-BMI. [file 12933_2023_2114_MOESM2_ESM.pdf]
