# Supplementary material for: Association between the cumulative average triglyceride glucose-body mass index and cardiovascular disease incidence among the middle-aged and older population: a prospective nationwide cohort study in China
Source: Cardiovasc Diabetol. 2024 Jan 6;23:16. doi: 10.1186/s12933-023-02114-w (PMC10771655; doi:10.1186/s12933-023-02114-w)
Supplement: Supplementary file 3 — Additional file 3: Table S1. Collinearity Statistics. Table S2. Baseline characteristics of individuals classified by outcome. Table S3. Association between the cumulative average TyG-BMI and the components of CVD. Table S4. Association between the cumulative average TyG-BMI and CVD incidence after excluding individuals with any missing value. Table S5. Association between the cumulative average TyG-BMI and CVD incidence after excluding individuals with CVD at Wave 2. Table S6. Baseline characteristics before and after 1:1 propensity score matching. Table S7. Association between the cumulative average TyG-BMI and CVD incidence after 1:1 propensity score matching. [file 12933_2023_2114_MOESM3_ESM.docx]

**Table S1.** Collinearity Statistics

| **Variables** | **GVIF** | **Df** | **GVIF^1/2Df^** |
| --- | --- | --- | --- |
| Cumulative average TyG-BMI | 1.760099 | 1 | 1.326687 |
| Age | 1.46553 | 1 | 1.210591 |
| Gender | 2.417298 | 1 | 1.554766 |
| Residence | 1.206166 | 1 | 1.098256 |
| Education level | 1.408403 | 2 | 1.089386 |
| Marital status | 1.134802 | 1 | 1.065271 |
| SBP | 1.874856 | 1 | 1.369254 |
| DBP | 1.882927 | 1 | 1.372198 |
| Smoking status | 2.112019 | 2 | 1.20552 |
| Drinking status | 1.483656 | 2 | 1.103655 |
| HbA1c | 1.343761 | 1 | 1.159207 |
| TC | 3.826225 | 1 | 1.956074 |
| HDL-c | 1.456616 | 1 | 1.206903 |
| LDL-c | 3.461869 | 1 | 1.86061 |
| Liver disease | 1.025241 | 1 | 1.012542 |
| Kidney disease | 1.01662 | 1 | 1.008276 |
| Diabetes | 1.811747 | 1 | 1.346012 |
| Hypoglycemic treatment | 1.693794 | 1 | 1.301458 |
| Dyslipidemia | 2.503335 | 1 | 1.582193 |
| Lipid-lowering treatment | 2.401454 | 1 | 1.549663 |
| Hypertension | 2.344611 | 1 | 1.531212 |
| Antihypertensive treatment | 1.681961 | 1 | 1.296904 |

Abbreviations: TyG-BMI, triglyceride glucose-body mass index; SBP, systolic blood pressure; DBP, diastolic blood pressure; HbA1c, glycosylated hemoglobin A1c; TC, total cholesterol; HDL‐c, high‐density lipoprotein cholesterol; LDL-c, low-density lipoprotein cholesterol.

**Table S2.** Baseline characteristics of individuals classified by outcome

| **Characteristics** | **Non - CVD** | **CVD** | **P-value** |
| --- | --- | --- | --- |
| n | 4875 | 543 |  |
| Gender (Female) | 2586 (53.0%) | 318 (58.6%) | 0.014 |
| Age, years | 59.4 ± 8.8 | 61.6 ± 8.8 | <0.001 |
| Residence (Urban) | 651 (13.4%) | 96 (17.7%) | 0.006 |
| Education level |  |  | 0.078 |
| Elementary school or below | 3444 (70.6%) | 389 (71.6%) |  |
| Middle school | 1320 (27.1%) | 134 (24.7%) |  |
| College or above | 111 (2.3%) | 20 (3.7%) |  |
| Current married | 4374 (89.7%) | 464 (85.5%) | 0.002 |
| SBP, mmHg | 129.3 ± 24.8 | 135.5 ± 26.6 | <0.001 |
| DBP, mmHg | 75.2 ± 11.9 | 77.6 ± 13.0 | <0.001 |
| BMI, kg/m^2^ | 23.3 ± 3.4 | 24.0 ± 3.6 | <0.001 |
| Smoking status |  |  | <0.001 |
| Never | 2969 (60.9%) | 345 (63.5%) |  |
| Former | 384 (7.9%) | 65 (12.0%) |  |
| Current | 1522 (31.2%) | 133 (24.5%) |  |
| Drinking status |  |  | 0.007 |
| Never | 2829 (58.0%) | 345 (63.5%) |  |
| Former | 273 (5.6%) | 37 (6.8%) |  |
| Current | 1773 (36.4%) | 161 (29.7%) |  |
| Dyslipidemia | 326 (6.7%) | 88 (16.2%) | <0.001 |
| Hypertension | 1736 (35.6%) | 298 (54.9%) | <0.001 |
| Diabetes | 717 (14.7%) | 102 (18.8%) | 0.012 |
| Liver disease | 160 (3.3%) | 29 (5.3%) | 0.013 |
| Kidney disease | 261 (5.4%) | 42 (7.7%) | 0.022 |
| Lipid-lowering treatment | 164 (3.4%) | 56 (10.3%) | <0.001 |
| Antihypertensive treatment | 688 (14.1%) | 167 (30.8%) | <0.001 |
| Hypoglycemic treatment | 211 (4.3%) | 44 (8.1%) | <0.001 |
| FBG, mg/dL | 109.1 ± 34.3 | 111.6 ± 35.6 | 0.120 |
| HbA1c, % | 5.2 ± 0.8 | 5.3 ± 0.8 | 0.021 |
| TG, mg/dL | 131.1 ± 104.7 | 134.1 ± 80.8 | 0.528 |
| TC, mg/dL | 192.9 ± 37.6 | 197.5 ± 38.3 | 0.008 |
| HDL-c, mg/dL | 51.5 ± 15.3 | 51.4 ± 15.7 | 0.895 |
| LDL-c, mg/dL | 115.7 ± 34.5 | 120.1 ± 35.6 | 0.005 |
| TyG | 8.7 ± 0.7 | 8.7 ± 0.6 | 0.009 |
| Cumulative average TyG-BMI | 204.1 ± 35.5 | 212.4 ± 36.7 | <0.001 |

Note: Variables are presented as mean ± SD or n (%).

Abbreviations: CVD, cardiovascular disease; SBP, systolic blood pressure; DBP, diastolic blood pressure; BMI, body mass index; FBG, fasting blood glucose; HbA1c, glycosylated hemoglobin A1c; TG, triglyceride; TC, total cholesterol; HDL‐c, high‐density lipoprotein cholesterol; LDL-c, low-density lipoprotein cholesterol; TyG, triglyceride glucose index; TyG-BMI, triglyceride glucose-body mass index.

**Table S3.** Association between the cumulative average TyG-BMI and the components of CVD

| **Cumulative Average TyG-BMI** | **Quartiles** | | | | | **Continuous** |
| --- | --- | --- | --- | --- | --- | --- |
|  | **Quartile 1** | **Quartile 2** | **Quartile 3** | **Quartile 4** | **P for trend** | **Per 1 SD increase** |
| Heart Condition |  |  |  |  |  |  |
| Cases, n (%) | 77 (5.7%) | 110 (8.1%) | 135 (10.0%) | 148 (10.9%) | - | - |
| Crude, OR (95%CI) | Reference | 1.468 (1.088-1.990) | 1.838 (1.378-2.467) | 2.035 (1.533-2.721) | <0.001 | 1.284 (1.171-1.408) |
| Model 1, OR (95%CI) | Reference | 1.562 (1.154-2.124) | 2.001 (1.492-2.701) | 2.255 (1.682-3.045) | <0.001 | 1.330 (1.209-1.462) |
| Model 2, OR (95%CI) | Reference | 1.603 (1.176-2.195) | 2.077 (1.519-2.858) | 2.375 (1.684-3.368) | <0.001 | 1.373 (1.222-1.542) |
| Model 3, OR (95%CI) | Reference | 1.519 (1.111-2.085) | 1.791 (1.301-2.479) | 1.790 (1.252-2.571) | 0.003 | 1.225 (1.084-1.384) |
| Stroke |  |  |  |  |  |  |
| Cases, n (%) | 20 (1.5%) | 23 (1.7%) | 28 (2.1%) | 24 (1.8%) | - | - |
| Crude, OR (95%CI) | Reference | 1.153 (0.630-2.128) | 1.410 (0.794-2.547) | 1.204 (0.662-2.210) | 0.486 | 1.053 (0.859-1.284) |
| Model 1, OR (95%CI) | Reference | 1.307 (0.711-2.424) | 1.699 (0.947-3.102) | 1.569 (0.846-2.941) | 0.121 | 1.159 (0.940-1.421) |
| Model 2, OR (95%CI) | Reference | 1.164 (0.624-2.189) | 1.316 (0.704-2.501) | 1.003 (0.486-2.087) | 0.993 | 0.964 (0.745-1.241) |
| Model 3, OR (95%CI) | Reference | 1.119 (0.597-2.112) | 1.234 (0.650-2.377) | 0.835 (0.389-1.798) | 0.626 | 0.890 (0.678-1.162) |

Note: Model 1, adjusted for age and gender; Model 2, adjusted for age, gender, smoking status, drinking status, SBP, DBP, HbA1c, TC, HDL-c, LDL-c; Model 3, adjusted for variables included in Model 2 and residence (hukou), education level, marital status, hypertension, dyslipidemia, diabetes, liver disease, kidney disease, antihypertensive treatment, lipid-lowering treatment and hypoglycaemic treatment.

Abbreviations: TyG-BMI, triglyceride glucose-body mass index; OR, odds ratio; CI, confidence interval; SD, standard deviation.

**Table S4.** Association between the cumulative average TyG-BMI and CVD incidence after excluding individuals with any missing value

| **Cumulative Average TyG-BMI** | **Quartiles** | | | | | **Continuous** |
| --- | --- | --- | --- | --- | --- | --- |
|  | **Quartile 1** | **Quartile 2** | **Quartile 3** | **Quartile 4** | **P for trend** | **Per 1 SD increase** |
| Median | 164.6 | 190.2 | 213.3 | 249.0 | - | - |
| Cases, n (%) | 90 (6.8) | 127 (9.7) | 148 (11.3) | 166 (12.6) | - | - |
| Crude, OR (95%CI) | Reference | 1.455 (1.100-1.933) | 1.726 (1.315-2.277) | 1.965 (1.506-2.579) | <0.001 | 1.270 (1.163-1.386) |
| Model 1, OR (95%CI) | Reference | 1.561 (1.176-2.081) | 1.902 (1.441-2.523) | 2.209 (1.676-2.929) | <0.001 | 1.322 (1.208-1.447) |
| Model 2, OR (95%CI) | Reference | 1.553 (1.162-2.083) | 1.866 (1.388-2.521) | 2.088 (1.510-2.901) | <0.001 | 1.310 (1.172-1.464) |
| Model 3, OR (95%CI) | Reference | 1.489 (1.111-2.003) | 1.644 (1.215-2.233) | 1.582 (1.127-2.228) | 0.020 | 1.169 (1.040-1.314) |

Note: Model 1, adjusted for age and gender; Model 2, adjusted for age, gender, smoking status, drinking status, SBP, DBP, HbA1c, TC, HDL-c, LDL-c; Model 3, adjusted for variables included in Model 2 and residence (hukou), education level, marital status, hypertension, dyslipidemia, diabetes, liver disease, kidney disease, antihypertensive treatment, lipid-lowering treatment and hypoglycaemic treatment.

Abbreviations: TyG-BMI, triglyceride glucose-body mass index; OR, odds ratio; CI, confidence interval; SD, standard deviation.

**Table S5.** Association between the cumulative average TyG-BMI and CVD incidence after excluding individuals with CVD at Wave 2

| **Cumulative Average TyG-BMI** | **Quartiles** | | | | | **Continuous** |
| --- | --- | --- | --- | --- | --- | --- |
|  | **Quartile 1** | **Quartile 2** | **Quartile 3** | **Quartile 4** | **P for trend** | **Per 1 SD increase** |
| Median | 164.6 | 190.1 | 213.4 | 248.9 | - | - |
| Cases, n (%) | 64 (4.9) | 92 (7.0) | 112 (8.5) | 122 (9.3) | - | - |
| Crude, OR (95%CI) | Reference | 1.472 (1.062-2.051) | 1.820 (1.330-2.510) | 1.999 (1.468-2.746) | <0.001 | 1.293 (1.170-1.429) |
| Model 1, OR (95%CI) | Reference | 1.535(1.105-2.146) | 1.934(1.405-2.682) | 2.148(1.562-2.980) | <0.001 | 1.328 (1.197-1.471) |
| Model 2, OR (95%CI) | Reference | 1.512 (1.080-2.127) | 1.872 (1.333-2.648) | 2.013 (1.387-2.940) | <0.001 | 1.321 (1.164-1.498) |
| Model 3, OR (95%CI) | Reference | 1.429 (1.019-2.017) | 1.630 (1.153-2.319) | 1.547 (1.051-2.290) | 0.050 | 1.191 (1.043-1.360) |

Note: Model 1, adjusted for age and gender; Model 2, adjusted for age, gender, smoking status, drinking status, SBP, DBP, HbA1c, TC, HDL-c, LDL-c; Model 3, adjusted for variables included in Model 2 and residence (hukou), education level, marital status, hypertension, dyslipidemia, diabetes, liver disease, kidney disease, antihypertensive treatment, lipid-lowering treatment and hypoglycaemic treatment.

Abbreviations: TyG-BMI, triglyceride glucose-body mass index; OR, odds ratio; CI, confidence interval; SD, standard deviation.

**Table S6.** Baseline characteristics before and after 1:1 propensity score matching

| **Covariates** | **Before Matching** | | |  | **After Matching** | | |
| --- | --- | --- | --- | --- | --- | --- | --- |
|  | **Q1-Q2** | **Q3-Q4** | **SMD** |  | **Q1-Q2** | **Q3-Q4** | **SMD** |
| n | 2709 | 2709 |  |  | 1355 | 1355 |  |
| Gender (Female) | 1249 (46.1) | 1655 (61.1) | 0.307 |  | 763 (56.3) | 758 (55.9) | -0.008 |
| Age, years | 61.02 (9.11) | 58.26 (8.23) | -0.335 |  | 59.21 (8.64) | 59.39 (8.71) | 0.021 |
| Residence (Urban) | 278 (10.3) | 469 (17.3) | 0.186 |  | 188 (13.9) | 183 (13.5) | -0.010 |
| Education level |  |  |  |  |  |  |  |
| Elementary school or below | 2004 (74.0) | 1829 (67.5) | -0.138 |  | 933 (68.9) | 951 (70.2) | 0.028 |
| Middle school | 653 (24.1) | 801 (29.6) | 0.120 |  | 390 (28.8) | 375 (27.7) | -0.024 |
| College or above | 52 (1.9) | 79 (2.9) | 0.059 |  | 32 (2.4) | 29 (2.1) | -0.013 |
| Current married | 2359 (87.1) | 2479 (91.5) | 0.159 |  | 1217 (89.8) | 1211 (89.4) | -0.016 |
| SBP, mmHg | 126.19 (22.73) | 133.62 (26.60) | 0.279 |  | 129.35 (25.67) | 129.49 (23.39) | 0.005 |
| DBP, mmHg | 72.82 (11.66) | 78.11 (11.89) | 0.445 |  | 75.34 (12.20) | 75.23 (10.94) | -0.009 |
| Smoking status |  |  |  |  |  |  |  |
| Never | 1460 (53.9) | 1854 (68.4) | 0.313 |  | 860 (63.5) | 844 (62.3) | -0.025 |
| Former | 213 (7.9) | 236 (8.7) | 0.030 |  | 109 (8.0) | 104 (7.7) | -0.013 |
| Current | 1036 (38.2) | 619 (22.8) | -0.367 |  | 386 (28.5) | 407 (30.0) | 0.037 |
| Drinking status |  |  |  |  |  |  |  |
| Never | 1480 (54.6) | 1694 (62.5) | 0.163 |  | 822 (60.7) | 818 (60.4) | -0.006 |
| Former | 155 (5.7) | 155 (5.7) | 0.000 |  | 74 (5.5) | 74 (5.5) | 0.000 |
| Current | 1074 (39.6) | 860 (31.7) | -0.170 |  | 459 (33.9) | 463 (34.2) | 0.006 |
| Dyslipidemia | 94 (3.5) | 320 (11.8) | 0.258 |  | 74 (5.5) | 82 (6.1) | 0.018 |
| Hypertension | 756 (27.9) | 1278 (47.2) | 0.386 |  | 480 (35.4) | 469 (34.6) | -0.016 |
| Diabetes | 260 (9.6) | 559 (20.6) | 0.273 |  | 169 (12.5) | 165 (12.2) | -0.007 |
| Liver disease | 94 (3.5) | 95 (3.5) | 0.002 |  | 48 (3.5) | 41 (3.0) | -0.028 |
| Kidney disease | 164 (6.1) | 139 (5.1) | -0.042 |  | 67 (4.9) | 77 (5.7) | 0.033 |
| Lipid-lowering treatment | 43 (1.6) | 177 (6.5) | 0.200 |  | 36 (2.7) | 36 (2.7) | 0.000 |
| Antihypertensive treatment | 225 (8.3) | 630 (23.3) | 0.354 |  | 179 (13.2) | 156 (11.5) | -0.040 |
| Hypoglycemic treatment | 71 (2.6) | 184 (6.8) | 0.166 |  | 48 (3.5) | 45 (3.3) | -0.009 |
| HbA1c, % | 5.14 (0.61) | 5.36 (0.91) | 0.238 |  | 5.19 (0.71) | 5.21 (0.58) | 0.019 |
| TC, mg/dL | 187.91 (36.16) | 198.88 (38.48) | 0.285 |  | 191.14 (37.24) | 192.37 (35.70) | 0.032 |
| HDL-c, mg/dL | 56.88 (15.71) | 46.04 (12.86) | -0.843 |  | 50.96 (12.58) | 50.77 (13.39) | -0.015 |
| LDL-c, mg/dL | 113.30 (31.91) | 119.07 (36.90) | 0.156 |  | 118.09 (33.64) | 118.41 (32.37) | 0.009 |

Note: Variables are presented as mean ± SD or n (%).

Abbreviations: Q1, Quartile 1; Q2, Quartile 2; Q3, Quartile 3; Q4, Quartile 4; SMD, standardized mean difference; SBP, systolic blood pressure; DBP, diastolic blood pressure; HbA1c, glycosylated hemoglobin A1c; TC, total cholesterol; HDL‐c, high‐density lipoprotein cholesterol; LDL-c, low-density lipoprotein cholesterol.

**Table S7.** Association between the cumulative average TyG-BMI and CVD incidence after 1:1 propensity score matching

| **Cumulative Average TyG-BMI** | **Cases, n (%)** | **OR (95% CI) *** | **P-value** |
| --- | --- | --- | --- |
| Categories |  |  |  |
| Quartile 1-2 | 121 (8.9) | Reference | - |
| Quartile 3-4 | 157 (11.6) | 1.337 (1.042-1.719) | 0.023 |
| Continuous |  |  |  |
| Per 1 SD increase | - | 1.190 (1.054-1.342) | 0.005 |

Note: *The odds ratios (OR) with their 95% confidence intervals (CI) for univariable logistic regression model were presented since no further covariate adjustments were required after 1:1 propensity score matching (PSM).

Abbreviations: TyG-BMI, triglyceride glucose-body mass index; OR, odds ratio; CI, confidence interval; SD, standard deviation.
